# Non-medical Use of Prescription Stimulants Among College Students: Non-oral Routes of Administration, Risk Factors, Motivations, and Pathways

**DOI:** 10.3389/fpsyt.2021.667118

**Published:** 2021-08-16

**Authors:** Stephen F. Butler, Stephen V. Faraone, Anthony L. Rostain, Jeffrey H. Newcorn, Kevin M. Antshel, Rebekkah S. Robbins, Jody L. Green

**Affiliations:** ^1^Inflexxion, an IBH Company, Irvine, CA, United States; ^2^Departments of Psychiatry and of Neuroscience and Physiology, The State University of New York (SUNY) Upstate Medical University, Syracuse, NY, United States; ^3^Department of Psychiatry, Cooper Medical School of Rowan University, Camden, NJ, United States; ^4^Department of Psychiatry, Icahn School of Medicine at Mount Sinai, New York, NY, United States; ^5^Department of Psychology, Syracuse University, Syracuse, NY, United States

**Keywords:** attention deficit/hyperactivity disorder, college students, non-medical use of prescription stimulants, self-report, substance related disorders

## Abstract

**Introduction:** Non-medical use (NMU) of prescription stimulant medications is a continuing public health concern. Stimulant medications prescribed for attention-deficit/hyperactivity disorder (ADHD) are widely available on college campuses, and, as a consequence, college students may have multiple opportunities to engage in prescription stimulant NMU. This online self-report survey examined prescription stimulant NMU among college students, including: (1) patterns of non-oral route of administration (ROA); (2) motivations for non-oral ROAs; and (3) retrospectively recalled pathways of initiation.

**Method:** The survey sample was created from a pool of 3,379 respondents, who were matched to a sampling frame constructed from the 18–26-year-old, college student sample of the 2016 American Community Survey (ACS). About 14% (*n* = 486) from the overall pool were identified as college students with self-reported prescription stimulant NMU, all of whom completed the survey. The survey covered user characteristics, prescription and illicit substance use, age of first NMU, motivations for NMU, sources of procurement, and ROAs used.

**Results:** Among 486 students reporting prescription stimulant NMU, 43% had a lifetime diagnosis of ADHD. More than 90% reported polysubstance use, with 55% using illicit substances other than marijuana. Slightly more than 2 in 5 (43.3%) reported using illicit substances prior to prescription stimulant NMU, 24.6% used both at the same age, and 32.0% engaged in NMU of prescription stimulants prior to using illicit substances. Prescription stimulant NMU preceded prescription opioid NMU 45% of the time. More than a quarter of those engaged in prescription stimulant NMU (27.9%) initiated prescription stimulants alone or at the same age as other drugs. Most prescription stimulant NMU was oral, however 23.0% reported any non-oral use: snorting (20.4%), smoking (6.0%)and/or injection (3.5%). Non-oral use was associated with being male, obtaining medication from a dealer, use to get high, and/or a substance use disorder diagnosis.

**Conclusions:** Prescription stimulant NMU often occurs in the larger context of other substance use among college students. Injection, an under-researched route for prescription stimulants, was associated with male gender, history of substance use and higher likelihood of illicit substance use. Nearly a quarter of college student survey respondents reported use with non-oral routes, which is associated with other high-risk behaviors. Efforts to reduce non-oral prescription stimulant NMU in college students are warranted.

## Introduction

Non-medical use (NMU) of prescription stimulant medications is a continuing public health concern (1–3). Emergency department (ED) visits related to NMU of stimulant medications increased 200% from 2005 to 2010 (4). Between 2006 and 2011, despite no change in treatment visits for adults involving prescriptions of dextroamphetamine, amphetamine or methylphenidate, NMU of prescription stimulants increased 67% and ED visits increased 156% (5). College students are particularly likely to engage in NMU of prescription stimulants (5–10). Past-year NMU of prescription stimulants is more common among 18–25 year-olds (~7.5%) compared with younger and older age groups (both ≤ 2%) (11).

Stimulant medications prescribed for attention-deficit/hyperactivity disorder (ADHD) are widely available on college campuses (12). One longitudinal study (13, 14) followed a single cohort of college freshmen for 4 years. Over the follow-up period, nearly two-thirds were offered prescription stimulants for NMU and 31% reported NMU, a prevalence echoed by other surveys (15).

One motivation for stimulant NMU is enhanced academic performance (1, 16), despite evidence that stimulant NMU does not improve academic performance (7, 10, 17). Stimulant NMU is associated with other drug use and/or heavy drinking (18, 19).

While most stimulant NMU is by oral routes, non-oral NMU has been noted (6, 14, 20). Only a few studies of college students have evaluated non-oral ROAs of prescription stimulants (14, 20), usually snorting and less often injection and smoking (7, 21). Drugs taken via injection, smoking and snorting have a more rapid onset than oral ingestion, resulting in greater reinforcement for getting high and addictive potential (22). Non-oral routes also enhance the risk of cardiovascular failure, cardiac arrhythmia, high blood pressure, paranoia (10) as well as hospitalization and death (23). Levels of prescription stimulant smoking or injection (when reported) in the general population of adults (6) and among college students (20) have been low (<6%). Estimates of snorting by college students range from 17% (14) to ~40% (20, 21).

The Food and Drug Administration (FDA) has declared NMU of prescription stimulants a serious public health concern and has called for the assessment of the potential impact of abuse deterrent formulations (ADFs) for prescription stimulants (24). ADFs, designed to deter non-oral use, have demonstrated benefits in reducing abuse of prescription drugs and associated adverse consequences, especially for prescription opioids (25–27). Examination of non-oral ROAs of prescription stimulants by college students has been previously identified as useful for determining the potential value of abuse deterrent formulations (ADFs) (16, 19). Surprisingly, little research has considered the characteristics or practices of college students who use non-oral ROAs.

One such characteristic is polydrug use, which is the misuse of more than one class of psychoactive prescription drugs (28). Toward this end we examined prescription stimulant NMU in the context of polydrug use by college students. We sought to investigate, descriptively, pathways of non-medical use and abuse of other non-alcohol substances. This may be particularly important given that initial exposure to prescription stimulants may occur via legitimate treatment for ADHD symptoms, as opposed to the traditional “gateway hypothesis” (29) which posits that early experimentation with alcohol, tobacco or marijuana is associated in a causal way to increased substance involvement later in life.

The present cross-sectional study used a self-report, online survey to characterize a sample of college students reporting NMU of prescription stimulants to gain a preliminary view of respondents' routes of administration, factors contributing to the NMU, and pathways of initiation associated with specific patterns of non-oral use. We particularly sought to consider whether prescription stimulant NMU by college students is the only type of misuse/abuse of non-alcohol substances, is an initial foray into misuse/abuse of other substances or occurs within the context of ongoing polysubstance abuse. Finally, we sought to examine the associations among different ROAs, motivations for NMU and sources of procurement of the prescription stimulants being misused.

## Methods

### Definition of Non-medical Use and Non-oral Use

Following the convention employed by the National Survey on Drug Use and Health (NSDUH) (30), medication NMU was defined as meeting at least one of three criteria within the respondents' lifetime: (1) use for any reason, even once, without one's own prescription; (2) use in ways other than prescribed, such as taking more than prescribed or more often than prescribed; and (3) use for the feeling or experience that the medication caused, such as feeling high, enhancement of other drugs, or prevention or treatment of withdrawal symptoms or other feelings. Non-oral use was defined as use by smoking, snorting and/or injection (31). Oral use included swallowing whole, chewing, and dissolving in liquid then swallowing.

### Study Population

An online survey of college students was conducted using an opt-in panel operated by YouGov^®^, an Internet-based market research and data analytics firm. The sample was created by YouGov from a pool of 3,379 respondents, who were matched to a sampling frame constructed from the 18–26-year-old, college student sample of the 2016 American Community Survey (ACS). The matched cases were weighted to the sampling frame using propensity scores based on age, gender, race/ethnicity, years of education, and region. The current study includes a subsample of 486 respondents who met inclusion criteria (age 18–26 years, currently enrolled in college, and reported prescription stimulant NMU at any time in their lifetime).

Participants are not paid to join the YouGov panel but receive points for completing surveys that can be redeemed for cash or charitable contributions. All participants provided electronic informed consent, however, respondents remained anonymous to the researchers. The consent form and study received approval by the New England Institutional Review Board (NEIRB).

### Questionnaire

The author-constructed survey took <15 min to complete. Respondents provided demographic information, college characteristics, and medical history, including self-report of lifetime substance use disorder (SUD) and psychiatric diagnoses. All participants reported lifetime prescription stimulant NMU and were asked about the time frame of use, motivations for NMU, sources of medication procurement, and ROA. The survey was limited to Schedule II pharmaceutical products that are FDA approved for ADHD and are in tablet or capsule form intended for oral administration. Other products or compounds, such as the methylphenidate analogs (32), were not included as part of the survey. Respondents were asked about lifetime NMU of prescription opioids, binge alcohol use in the past 30 days (five or more drinks in a single occasion), lifetime marijuana use, and lifetime “illegal drug” use, including cocaine/crack, heroin, street fentanyl, inhalants, amphetamines/methamphetamines, hallucinogens, sedatives, or other illegal substances. Students estimated their age at first NMU of prescription drugs, marijuana, and illegal drugs, in order to calculate drug initiation pathways of prescription stimulant NMU for polysubstance users.

### Statistical Analysis

Sample characteristics are presented as frequencies and proportions. Self-reported pathways of initiation of prescription stimulant NMU are presented descriptively based on retrospective answers to the age-at-first-use question. Univariate pairwise comparisons of ROA subgroups (oral-only, snorting with no injection, and injection) used chi-square tests, Fisher's exact tests, or *t*-tests as appropriate.

Logistic regression models evaluated factors associated with non-oral vs. oral-only prescription stimulant NMU, including: age; sex; race; diagnoses of ADHD, lifetime psychiatric disorder (depression, anxiety or bipolar disorder), alcohol or substance use disorder, or conduct or oppositional defiant disorder; binge alcohol use in the past 30 days; lifetime marijuana use, lifetime use of cocaine, heroin, or illegal stimulant, and lifetime NMU of prescription opioids. Findings are presented as odds ratios (OR) and 95% confidence intervals (CI).

Statistical analyses were conducted using Statistical Analysis Software (SAS) Enterprise Guide Version 7.1 (SAS Institute, Cary, NC). For logistic regression models, *p*-values were two-sided and considered significant at *p*-values < 0.05. To explore correlates associated with various ROAs employed by participants, we conducted a high number of pairwise comparisons. Bonferroni adjusted *p*-values were used for analyses of medication procurement and motivation (*p* < 0.006). Exploratory analyses of factors associated with different ROA use employed an adjusted *p*-value of 0.003. Otherwise, *p*-values < 0.05 are presented in the tables.

## Results

### Survey Sample Characteristics

Among the 486 college students reporting lifetime prescription stimulant NMU, 59.9% were women; most were white, single, and full-time undergraduates from public schools (Table 1). Colleges were in the South (39.9%), Midwest (21.4%), West (20.8%) and Northeast (17.9%). Two-hundred-ten students (43.2%) reported a lifetime diagnosis of ADHD, of which 70% were diagnosed at age 16 or older. More than three-quarters (77.6%) reported a lifetime psychiatric diagnosis (depression, anxiety, or bipolar disorder), while 29.6% reported a lifetime alcohol or substance use disorder. Binge alcohol use in the past 30 days was reported by 12%, while lifetime use of cocaine, heroin and illicit stimulants ranged from 15.8% for heroin to 35.4% for cocaine. Lifetime NMU of prescription opioid medication was 58.6%, and marijuana use was reported by 73.7%.

**Table 1:** Characteristics of college students by lifetime non-medical use of prescription stimulants.

**Characteristic**	**Total sample (*N* = 486) No. (%)**
**Sex**	
Male	195 (40.1)
Female	291 (59.9)
**Age, years**	
18–20	162 (33.3)
21–23	196 (40.3)
24–26	128 (26.3)
**Race**	
White	319 (65.6)
Black	54 (11.1)
Hispanic/Latino	47 (9.7)
Asian	41 (8.4)
Native American	4 (0.8)
Middle Eastern	6 (1.2)
Other	2 (0.4)
Mixed race	13 (2.7)
**Marital status**	
Married/living with spouse	81 (16.7)
Separated	7 (1.4)
Divorced	4 (0.8)
Widowed	1 (0.2)
Single/never married	365 (75.1)
Domestic partnership	28 (5.8)
**Year of college**	
Undergraduate/community college	358 (73.7)
Graduate	128 (26.3)
**Type of student**	
Full-time	370 (76.1)
Part-time	116 (23.9)
**Type of college**	
Public	378 (77.8)
Private	108 (22.2)
**US Region for the college**	
Northeast Region	87 (17.9)
Midwest Region	104 (21.4)
South Region	194 (39.9)
West Region	101 (20.8)
Lifetime diagnosis of ADHD	210 (43.2)
Lifetime diagnosis of psychiatric disorder (depression, anxiety, or bipolar disorder)	377 (77.6)
Lifetime diagnosis of alcohol or substance use disorder	144 (29.6)
Lifetime diagnosis of conduct or oppositional defiant disorder	124 (25.5)
Binge alcohol use in past 30 days^a^	59 (12.1)
Marijuana use in lifetime	358 (73.7)
Cocaine use in lifetime	172 (35.4)
Heroin use in lifetime	77 (15.8)
Illicit stimulant use in lifetime	134 (27.6)
Non-medical use of prescription opioids in lifetime	285 (58.6)

*NMU, nonmedical use*.

a *Binge drinking defined as consuming five or more drinks in a single occasion*.

### Factors Associated With Use by Non-oral Routes

For the logistic regression and ROA analyses, 99.2% of all respondents (*n* = 482) were included in the analyses (four were excluded due to missing data). Among college students reporting prescription stimulant NMU included in the logistic regression analyses, 23.2% (*n* = 112) reported at least one non-oral ROA. Univariate results of the logistic regression (Table 2) identified factors associated with non-oral ROAs, including male gender (OR: 0.49, 95% CI: 0.32–0.076), lifetime diagnoses of alcohol or other substance use disorder (OR: 3.14, 95% CI: 2.02–4.88), conduct/oppositional defiant disorder (OR: 3.65, 95% CI: 2.32–5.74), and lifetime use of illegal drugs (i.e., cocaine, heroin or illegal stimulants; OR: 2.40, 95% CI:1.56–3.69). A lifetime diagnosis of ADHD (OR: 1.92, 95% CI: 1.26–2.95) and a history of NMU of prescription opioids (OR: 1.85, 95% CI: 1.18–2.90) were also significantly associated with use by a non-oral ROA, albeit to a lesser degree. Multivariable analyses yielded significant adjusted ORs for only two factors: lifetime diagnosis of conduct/oppositional defiant disorder (adjusted OR: 2.27, 95% CI: 1.15–4.48) and lifetime use of illicit drugs (adjusted OR: 1.99, 95% CI: 1.18–3.37) (Table 2), implying a relatively high concordance between many of the various factors investigated (*r*_s_ among the significant variables in the univariate analysis ranged from 0.17 to 0.66, all *p* < 0.001) (33).

**Table 2:** Factors associated with lifetime non-medical use of prescription stimulants via non-oral routes^*^ compared with oral routes only among college students (*N* = 482^**^).

	**Lifetime NMU of Rx stimulants via non-oral routes (** ***N*** **= 112)**		
	**No./Total (%)**	**Univariate-unadjusted OR (95% CI) *p*-value**	**Multivariable-adjusted OR (95% CI) *p*-value**
**Age, years**			
18–20	31/162 (19.1)	0.65 (0.38–1.14)	0.86 (0.47–1.58)
21–23	47/192 (24.5)	0.90 (0.54–1.50)	1.01 (0.58–1.74)
24–26	34/128 (26.6)	Ref	Ref
		NS	NS
**Sex**			
Female	52/288 (18.1)	0.49 (0.32–0.76)	0.65 (0.40–1.04)
Male	60/194 (30.9)	Ref	Ref
		*p* = 0.0012	NS
**Race/ethnicity**			
White	72/317 (22.7)	0.92 (0.59–1.43)	1.00 (0.62–1.62)
Non–white	40/165 (24.2)	Ref	Ref
		NS	NS
**Lifetime diagnosis of ADHD**			
Yes	62/207 (30.0)	1.92 (1.26–2.95)	1.02 (0.58–1.79)
No	50/275 (18.2)	Ref	Ref
		*p* = 0.003	NS
**Lifetime diagnosis of psychiatric disorder (depression, anxiety, or bipolar disorder)**			
Yes	94/374 (25.1)	1.68 (0.96–2.93)	1.13 (0.60–2.13)
No	18/108 (16.7)	Ref	Ref
		NS	NS
**Lifetime diagnosis of alcohol or substance use disorder**			
Yes	55/142 (38.7)	3.14 (2.02–4.88)	1.30 (0.66–2.55)
No	57/340 (16.8)	Ref	Ref
		*p* < 0.001	NS
**Lifetime diagnosis of conduct or oppositional defiant disorder**			
Yes	52/123 (42.3)	3.65 (2.32–5.74)	2.27 (1.15–4.48)
No	60/359 (16.7)	Ref	Ref
		*p* < 0.001	*p* = 0.019
**Binge alcohol use in past 30 days**			
Yes	16/58 (27.6)	1.30 (0.70–2.42)	1.48 (0.75–2.92)
No	96/424 (22.6)	Ref	Ref
		NS	NS
**Marijuana use in lifetime**			
Yes	81/355 (22.8)	0.92 (0.57–1.47)	0.90 (0.50–1.61)
No	31/127 (24.4)	Ref	Ref
		NS	NS
**Other illicit drug (cocaine, heroin, or illegal stimulant use) in lifetime**			
Yes	63/192 (32.8)	2.40 (1.56–3.69)	1.99 (1.18–3.37)
No	49/290 (16.9)	Ref	Ref
		*p* < 0.001	*p* < 0.010
**Non-medical use of prescription opioids in lifetime**			
Yes	78/283 (27.6)	1.85 (1.18–2.90)	1.09 (0.65–1.81)
No	34/199 (17.1)	Ref	Ref
		*p* = 0.008	NS

*CI, confidence interval; NMU, nonmedical use; OR, odds ratio; Ref, reference; Rx, prescription*.

* *Non-oral routes include snorting, smoking, and/or injection. Oral routes include swallowing whole, chewing in mouth then swallowing, and/or dissolving in liquid then swallowing*.

** *Four respondents were excluded from the model due to missing data*.

### Non-oral ROAs Reported

While most of the 486 college students reported NMU of stimulants via oral routes (92.0%), 23.0% reported any non-oral use: snorting (20.4%), smoking (6.0%) and/or injection (3.5%). Among those reporting non-oral NMU of prescription stimulants, two-thirds (66.1%) also reported oral NMU. Among the 112 non-oral users, 88.4% reported snorting, 25.9% reported smoking, and 15.2% reported injecting. Since individuals often report more than one ROA, percentages do not add to 100%.

### Source of Procurement for Prescription Stimulants

Overall, most respondents (67.3%) reported obtaining the medication from a family member or friend, followed by their own prescription (39.7%). Table 3 (top) presents comparisons of drug source in respondents who use oral routes only with those who snort but do not inject, and those who inject. Respondents reporting injecting and/or snorting were more likely than oral-only respondents to obtain their drug from a dealer, to have bought online without a prescription, or to have faked a prescription (*p* < 0.001). Report of injection was more often than oral-only use to be associated with stealing the drug (*p* = 0.004). Those who reported snorting were more likely than oral-only users to have traded for the drug (*p* = 0.001). Significant differences were not found for snorting only vs. injection.

**Table 3:** Source of procurement and motivations for NMU of prescription stimulants by route.

	**Oral-only *N* = 370**	Snorting no injection *N* = 88^*^	**Injection *N* = 17**	Oral-only vs. Snorting no injection^**^	Oral-only vs. injection^**^	Injection vs. Snorting no injection^**^
**Number and percent** ^¥^ **indicating source of procurement of prescription stimulant by route**						
Bought/given/stole it from family or friend	239 (64.6%)	70 (79.5%)	10 (58.8%)	0.008	NS	NS
My own prescription from one doctor or several doctors	146 (39.5%)	33 (37.5%)	11 (64.7%)	NS	0.045	NS
Bought it from a dealer	29 (7.8%)	26 (29.6%)	8 (47.1%)	<0.001	<0.001	NS
Bought it online without a doctor's visit	24 (6.5%)	19 (21.6%)	8 (47.1%)	<0.001	<0.001	0.037
Traded for it	11 (3.0%)	11 (12.5%)	2 (11.8%)	0.001	NS	NS
Wrote or bought a fake prescription	4 (1.1%)	9 (10.2%)	5 (29.4%)	<0.001	<0.001	0.049
Stole them from someone I did not know	5 (1.4%)	5 (5.7%)	3 (17.6%)	0.026	0.004	NS
“Other” source	2 (0.5%)	0 (0.0%)	0 (0.0%)	NS	NS	NS
**Number and percent** ^¥^ **motivations for NMU of prescription stimulants by route**						
To enhance performance at work or school	189 (51.1%)	36 (40.9%)	4 (23.5%)	NS	0.044	NS
For energy	76 (20.5%)	25 (28.4%)	9 (52.9%)	NS	0.004	NS
To treat ADHD	69 (18.6%)	9 (10.2%)	4 (23.5%)	NS	NS	NS
To improve my mood or elevate my spirit	52 (14.1%)	20 (22.7%)	7 (41.2%)	NS	0.008	NS
To get high/enhance effect of other drugs	32 (8.6%)	28 (31.8%)	9 (52.9%)	<0.001	<0.001	NS
To control appetite or for weight loss	21 (5.7%)	17 (19.3%)	6 (35.3%)	<0.001	<0.001	NS
To prevent or treat withdrawal symptoms	7 (1.9%)	4 (4.5%)	4 (23.5%)	NS	0.001	0.023
By mistake (such as forgot you already took it)	7 (1.9%)	4 (4.5%)	4 (23.5%)	NS	0.001	0.023
Other	6 (1.6%)	2 (2.3%)	0 (0%)	NS	NS	NS

¥ *Respondents select all that apply, so percentages do not add to 100%*.

* *Seven individuals selected smoke and/or “other route” but not snorting or injection*.

** *Difference between groups: bivariate comparisons used χ^2^ and Fisher's Exact Test where appropriate. All p-values are 2-tailed*.

### Motivations for NMU of Prescription Stimulants

Nearly half (47.7%) of all respondents listed enhancing work or school performance as a motivation for prescription stimulant NMU, 23.3% for increased energy, and 14.6% for getting high or enhancing the effect of other drugs. Comparisons across ROA patterns (Table 3 lower) revealed that those who snort and/or inject were more likely than oral-only users to report getting high, enhancing the effect of other drugs, and controlling appetite as motivations for NMU. Those injecting were more likely than oral-only users to report motivation for energy, to treat withdrawal symptoms, and to report using the drug by mistake (i.e., “forgot they already took it”).

### Factors Associated With Prescription Stimulant NMU Route of Administration

Oral ROA was reported by 92.0% of respondents: 83.4% swallowed whole, 14.8% by chewing, and 18.3% dissolved in liquid to drink. Non-oral NMU was observed in 23.0% of all respondents. Within each group, 20.4% of the whole sample and 88.4% of the non-oral sample reported snorting; 6.0% of the whole sample and 25.9% of the non-oral group smoked. Injection was reported by 3.5% of the whole group and 15.2% of the non-oral NMU respondents.

Comparing characteristics of individuals who use oral routes only with those who snort but do not inject and those who inject are presented in Table 4. Compared with respondents reporting oral routes only, college students who reported injecting stimulants were more likely to be men, to be a member of a Greek organization, to have an ADHD diagnosis, to have a lifetime substance use diagnosis, and to have a history of NMU of prescription opioids. Those who injected were more likely than the oral-only group and the snorting/no injection group to report use of heroin, sedatives, and street fentanyl. Snorting/no injection respondents more often reported a substance use diagnosis and use of cocaine/crack than those reporting oral ROA only.

**Table 4:** Exploratory factors associated with ROA patterns reported for prescription stimulants.

	**Oral-only *N* = 370**	Snorting no injection *N* = 88^*^	**Injection *N* = 17**	Oral-only vs. Snorting no injection^**^	Oral-only vs. injection^**^	Injection vs. Snorting no injection^**^
Age, mean (SD)	21.7 (2.5)	22.0 (2.5)	22.2 (2.2)	NS	NS	NS
Race (White), No. (%)	245 (66.2)	60 (68.2)	10 (58.8)	NS	NS	NS
Gender (M), No. (%)	134 (36.2)	42 (47.7)	13 (76.5)	NS	<0.001	0.04
Member fraternity or sorority, No. (%)	100 (27.0)	33 (37.5)	11 (64.7)	NS	0.002	NS
Lifetime psychiatric, No. (%)	280 (75.7)	71 (80.7)	17 (100.0)	NS	0.02	NS
ADHD diagnosis, No. (%)	145 (39.2)	43 (48.9)	13 (76.5)	NS	0.002	NS
Substance use diagnosis, No. (%)	87 (23.5)	34 (38.6)	15 (88.2)	<0.001	<0.001	<0.001
Past 30-day Rx stimulant NMU, No. (%)	159 (43.0)	44 (50.0)	11 (64.7)	NS	NS	NS
NMU of prescription opioids, No. (%)	205 (55.4)	56 (63.6)	16 (94.1)	NS	0.002	0.01
Marijuana, No. (%)	274 (74.1)	66 (75.0)	11 (64.7)	NS	NS	NS
Cocaine or crack, No. (%)	109 (29.5)	48 (54.5)	10 (58.8)	<0.001	0.01	NS
Illicit amphetamines/methamphetamine, No. (%)	89 (24.1)	31 (35.2)	9 (52.9)	0.04	0.02	NS
Hallucinogens, No. (%)	117 (31.6)	40 (45.5)	10 (58.8)	0.02	0.02	NS
Heroin, No. (%)	49 (13.2)	15 (17.0)	9 (52.9)	NS	<0.001	0.003
Street fentanyl, No. (%)	39 (10.5)	11 (12.5)	10 (58.8)	NS	<0.001	<0.001
Inhalants, No. (%)	65 (17.6)	21 (23.9)	9 (52.9)	NS	0.001	0.02
Sedatives, No. (%)	49 (13.2)	17 (19.3)	10 (58.8)	NS	<0.001	0.002
Other illicit substance, No. (%)	45 (12.2)	15 (17.0)	6 (35.3)	NS	0.02	NS

* *Note: Seven individuals selected smoke and/or “other route” but not snorting or injection*.

** *Difference between groups: bivariate comparisons used χ^2^ and Fisher's Exact Test where appropriate. All p-values are 2-tailed*.

### Pathways of Initiation

Most respondents (*n* = 444, 91.4%) reported non-alcohol polysubstance use. Seventeen percent (*n* = 76) of the polysubstance users started prescription stimulant NMU at an age earlier than any of the other drugs endorsed. Another 12.2% (*n* = 54) first engaged in prescription stimulant NMU at the same age as at least one of the other drug categories. While the majority of those reporting polysubstance use also reported abusing other drugs before prescription stimulant NMU, 27.9% either initiated prescription stimulant NMU first or initiated polysubstance use concurrently with prescription stimulant NMU.

Table 5 presents an overview of the three sequences of misuse/abuse of prescription stimulants and other drugs for all polysubstance users and by ROA - i.e., prior to, coincident with, or following first NMU of prescription stimulants. Nearly two-thirds (64.2%) of all polysubstance users reported NMU of prescription opioids, of whom 38.6% initiated prescription opioid NMU prior to NMU of prescription stimulants, 16.1% initiated NMU of these medications at the same age, and 45.3% started NMU of prescription stimulants first. Among those who used illicit drugs other than marijuana (55.0% of all polysubstance users), 43.3% used such drugs prior to prescription stimulants, 24.6% at the same age, and 32.0% started NMU of prescription stimulants prior to using illicit drugs.

**Table 5:** Pathways of multi-drug use that involved prescription stimulant NMU^*^ for all respondents and by ROA.

**Polysubstance users^¥^**	**Total** ***N*** **= 444**		**Oral-only** ***N*** **= 370**		**Snorting no injection** ***N*** = 88^*^		**Injection** ***N*** **= 17**		Oral-only vs. Snorting no injection^**^	Oral-only vs. injection^**^	**Injection vs. Snorting** no injection^**^
	***n***	**Col %**	***n***	**Col %**	***n***	**Col %**	***n***	**Col %**	***p*-value**	***p*-value**	***p*-value**
**Prescription opioids**	**285**	**100.0**	**205**	**100.0**	**56**	**100.0**	**16**	**100.0**			
Before stimulants	110	38.6	73	35.6	25	44.6	8	50.0	NS	NS	NS
Same age stimulants	46	16.1	34	16.6	9	16.1	2	12.5			
After stimulants	129	45.3	98	47.8	22	39.3	6	37.5			
**Marijuana**	**358**	**100.0**	**274**	**100.0**	**66**	**100.0**	**11**	**100.0**			
Before stimulants	249	69.6	194	70.8	42	63.6	9	81.8	0.0489	NS	NS
Same age stimulants	38	10.6	24	8.8	13	19.7	0	0.0			
After stimulants	71	19.8	56	20.4	11	16.7	2	18.2			
**Illicit drugs (other than marijuana)^€^**	**244**	**100.0**	**171**	**100.0**	**57**	**100.0**	**11**	**100.0**			
Before stimulants	106	43.4	77	45.0	19	33.3	8	72.7	NS	NS	NS
Same age stimulants	60	24.6	40	23.4	18	31.6	1	9.1			
After stimulants	78	32.0	54	31.6	20	35.1	2	18.2			

¥ *Respondents reporting lifetime use of at least 1 of the following in addition to lifetime prescription stimulant NMU: prescription opioids, marijuana, or other illicit drugs (other than marijuana)*.

€ *Includes cocaine, heroin, street fentanyl, inhalants, illicit amphetamines, hallucinogens, barbiturates, and 'other' illicit substances*.

* *Seven individuals selected smoke and/or “other route” but not snorting or injection*.

** *Difference between groups: bivariate comparisons used χ^2^ and Fisher's Exact Test where appropriate. All p-values are 2-tailed*.

Lifetime marijuana use was reported by 80.6% of all polysubstance users, and 56.1% reported using marijuana or marijuana plus another substance prior to initiating prescription stimulant NMU. Among all marijuana users, ~70% reported using marijuana prior to prescription stimulant NMU, while nearly 20% reported NMU of prescription stimulants prior to marijuana use. Nearly 1 in 5 (18.7%) of all polysubstance users reported marijuana use and prescription stimulant NMU only, while 14.6% used marijuana prior to prescription stimulant NMU, 3.2% reported initiating prescription stimulant NMU prior to marijuana, and 0.9% initiated use of both substances at the same age.

The breakdown of the drug-use sequences in Table 5 by ROA pattern revealed no significant differences between those who report NMU of prescription stimulants orally only, those who snort but do not inject, and those who inject. While the percentages of those who used illicit drugs other than marijuana prior to NMU of prescription stimulants were much greater for those who inject (i.e., 45.0% for oral-only and 33.3% for snort/no inject vs. 72.7% for those who inject), these differences did not reach statistical significance.

## Discussion

This cross-sectional, online survey evaluated prescription stimulant NMU among college students in the United States. Pathways of initiation of prescription stimulant NMU were presented, as well as characteristics of non-oral use.

All respondents to this survey engaged in NMU of stimulants, while nearly one in four (23%) reported non-oral use. Non-oral use was consistently associated with increased likelihood of serious, non-alcohol substance use. Although other studies have found ADHD diagnosis, gender, race, depression, anxiety, marijuana and alcohol use to be associated with prescription stimulant NMU (34), in this study age, race, recent alcohol binge drinking, and marijuana use did not differentiate oral and non-oral NMU. Univariate analyses revealed associations of major factors with reports of non-oral ROA, especially being male, lifetime diagnoses of ADHD, alcohol or other substance disorder as well as conduct/oppositional defiant disorder, and history of prescription opioid NMU.

The finding that women students were less likely overall to report non-oral NMU practices, may be an underlying feature common to many of the other factors investigated. For instance, women are less likely to be diagnosed with ADHD and, women diagnosed with ADHD appear to exhibit lower levels of conduct problems than their male counterparts (35), both factors that were associated with non-oral ROA NMU in this survey. Women were significantly less likely to inject, even if they reported snorting prescription stimulants, however, oral-only ROA vs. snorting but not injection were not significantly different by gender. Injecting may be associated with higher risk-taking behavior in men with ADHD (35). On the other hand, other studies of gender differences in adults with ADHD (36) have found women with an ADHD diagnosis to be much more likely than women without one to express suicidal ideation, which may be a very different phenomenon than male risk-taking behavior.

Of those reporting non-oral prescription stimulant NMU, the large majority (88%) snorted the medication, with a quarter reporting smoking and 15% injecting. Our findings are consistent with those from the few studies investigating injection of prescription stimulants, suggesting that injection of these medications is relatively rare (6, 20, 23). Because the small number of college students who inject prescription stimulants in this sample resulted in point estimates that may not be stable, the findings reported for non-oral injection users should be considered preliminary and used to stimulate further research. However, even though injection may be rare, it is associated with much higher risk and understanding what motivates this behavior may inform prevention and treatment strategies.

Compared with oral-only users, respondents reporting any non-oral route were more likely to have more serious substance use histories and drug-use patterns. Non-oral use is consistently associated with serious adverse consequences (10), increased prevalence of overdose (37), addictive potential (38) and significant risk of morbidity and mortality (23). Note that in the current study, the large majority of those who reported NMU of prescription stimulants via non-oral routes snorted the drug. Further research into this type of non-oral prescription stimulant NMU is warranted, as such work may clarify a role that ADF formulations can have in reducing the serious adverse consequences of non-oral prescription stimulant NMU (16, 19, 23, 24).

Consistent with findings of earlier research (7, 10, 39), two-thirds of NMU respondents obtained the prescription stimulant from a family member or friend (either bought, given, or stolen). About 40% obtained the stimulant through a legitimate prescription. Non-oral users, on the other hand, endorsed other sources, especially getting the drug from a “dealer,” and online purchases, while also obtaining prescription stimulants through the usual sources of friends, family, and providers.

The motivation most often reported for NMU by oral routes and snorting was performance enhancement, as others have noted (3, 7, 10, 40). Such oral NMU of prescription stimulants for performance enhancement may signal the lack of adequate resources or supports for students in a highly artificial (e.g., memory-based performance testing), high stress environment, where academic outcomes can have life-altering consequences. NMU for performance enhancement by students with ADHD using oral routes also raises questions regarding the perceptions by these patients as to the adequacy of their treatment (16). It may also be relevant that learning problems often associated with an ADHD diagnosis may be more responsive to treatments other than medication (35). Although not among the most often reported motivations by those who inject, performance enhancement was reported as a motivation for NMU by nearly a quarter of those respondents. Those reporting snorting and injection were significantly more likely than the oral-only group to report motivations to get high or to enhance the effects of other drugs. These motivations and the higher likelihood of polysubstance use in non-oral users are indicative of higher risk behaviors often associated with having or developing significant substance use problems.

More than 90% of prescription stimulant NMU was observed in the context of polysubstance use (not including alcohol); a finding consistent with results of a population-based survey (41). Among polysubstance users, 18.7% reported marijuana as the only other drug used. While 80.6% of polysubstance users reported marijuana use, and 69.6% of those reported marijuana use prior to prescription stimulant NMU, marijuana was considered separately in this study, given its legal status in many states for use by at least some individuals in the examined age range as well as changing views of marijuana use by the public (42, 43). Nevertheless, it is noteworthy that prescription stimulant NMU preceded prescription opioid NMU 45% of the time and preceded illegal drug use 32% of the time. Our pathways analyses are conceptually similar to the so-called “gateway hypothesis” (29), which proposes that a child or adolescent's early experimentation with or exposure to alcohol, tobacco, or cannabis leads to more addictive illicit drugs later in adulthood (44, 45). Empirical examination of the gateway hypothesis has yielded conflicting and ambiguous results (45, 46), suggesting that a simple, causal role of early exposure to abusable or addictive substances is unlikely. Interpretation of early prescription stimulant NMU in the pathway toward use of illegal drugs, NMU of other prescription drugs or use by non-oral routes is complex. There is evidence from this survey and other research (47) that an ADHD diagnosis maybe a risk factor for later serious drug involvement (i.e., polysubstance use and non-oral ROA use). Specifically investigating exposure to ADHD medications in childhood and adolescence, McCabe et al. (48) found that children who initiated stimulant medication for ADHD early (aged 9 or less) and for longer duration (6 or more years) did not differ between population controls (youth without ADHD and unmedicated youth with ADHD).

The pathway findings among the 90% of survey respondents reporting polydrug use in this study support other studies that suggest primary care physicians largely underestimate the potential for NMU and diversion of ADHD medications by their patients (16). Thus, the findings presented here suggest that prescription stimulant NMU coincided with a more general pattern of substance misuse/abuse. Prescribers should at least be cognizant of the potential for current or later escalation of substance use by their patients. This study shares the limitations of other online surveys, including self-selection bias, self-report of drug use and medical diagnoses, and the use of an online panel. Although efforts were made to establish a representative sampling frame from which to draw respondents, the final sample may not be representative of college students who engage in NMU. While many of the findings are consistent with those from other studies, generalization should be approached cautiously. Use of a cross-sectional survey to explore retrospective recall of pathways of drug initiation across time is, by definition, limited, and can only be considered suggestive. In agreement with other authors (9), our use of the Internet panel survey is intended to complement, not replace, national probability studies.

## Conclusions

A number of authors have proposed policies and interventions to address college student prescription NMU that are often educational in nature, stressing legal and ethical concerns (i.e., is NMU “cheating?”) and combating the “misinformation” that prescription stimulant NMU is harmless (10). Over-estimation of the extent to which stimulant products enhance memory, attention and creativity for individuals outside the treatment of ADHD (49) may have important implications for educational interventions. Identifying and providing services to those at risk for mental health and substance use issues has also been proposed (10). A comprehensive strategic planning guide has recently been published online by the Drug Enforcement Administration (50) to address prescription stimulant NMU. The guide covers assessment, building capacity and programs, implementation, and evaluating the impact of interventions. Some authors have stressed viewing NMU as a “red flag” for possible involvement in other illicit drug use, poor academic performance, and mental health problems (18, 19) and called for better diagnostic practices for ADHD (7), which can present challenges in adults (51, 52).

Our finding that nearly a quarter of NMU involved non-oral routes highlights the importance of non-oral NMU of prescription stimulants. This concerning rate of non-oral NMU suggests that further work should explore methods to reduce such use and the deleterious outcomes associated with snorting or injecting the drugs. Promising efforts to reduce the prevalence and negative impacts of prescription stimulant NMU among college students likely include a range of preventive approaches addressing policy, education, diagnostic practices and detection of malingering, paired with treatment approaches utilizing cognitive behavior therapy (53, 54), interventions geared to patients' specific needs, such as personality-targeted interventions (55), sensitivity to the unique needs of women with ADHD, who, for instance, appear to be more impacted by parenting style (56) than male ADHD patients, use of therapies other than medications when learning problems are a major presenting complaint (35), and, possibly, medication formulations that present barriers to non-oral use (16, 19, 23, 24).

## Data Availability Statement

The raw data supporting the conclusions of this article will be made available by the authors, without undue reservation.

## Ethics Statement

The studies involving human participants were reviewed and approved by New England Institutional Review Board (NEIRB). The patients/participants provided electronic informed consent to participate in this study.

## Author Contributions

SB, SF, AR, JN, KA, and JG contributed to conception and design of the study. SB and RR performed the statistical analysis. SB wrote the first draft of the manuscript. SF, AR, JN, and JG wrote sections of the manuscript and provided specific edits/revisions. All authors contributed to the article and approved the submitted version.

## Conflict of Interest

SB is a consultant to Inflexxion, an IBH Company. Inflexxion contracts with FDA and multiple companies with interests in some of the products included in the compounds evaluated for this article. In the past year, SF received income, potential income, travel expenses continuing education support and/or research support from, Akili, Arbor, Genomind, Ironshore, Ondosis, Otsuka, Rhodes, Shire/Takeda, Sunovion, Supernus, Tris, and Vallon. With his institution, he has US patent US20130217707 A1 for the use of sodium-hydrogen exchange inhibitors in the treatment of ADHD. In previous years, he received support from: Alcobra, CogCubed, Eli Lilly, Enzymotec, Janssen, KemPharm, Lundbeck/Takeda, McNeil, Neurolifesciences, Neurovance, Novartis, Pfizer, and Vaya. In the past 2 years, JN is/has been an advisor and/or consultant for Adlon Therapeutics, Akili Interactive, Arbor, Enzymotec, Ironshore, Medice, NLS, OnDosis, Pfizer, Rhodes, Shire, and Supernus. He was a DSMB member for Pfizer and Sunovion, and received research funds from Enzymotec, Otsuka, Shire and Supernus. He also has received speaker fees from Shire for disease-state presentations and served as a consultant for the US National Football League. KA has current investigator-initiated research funding from Takeda Pharmaceutical Company and serves as an advisory board member for Arbor Pharmaceutical Company. AR reports book royalties (Routedge/Taylor Francis Group), scientific board honoraria (Shire/Takeda, Arbor), consultant honoraria (Tris Pharmaceuticals and National Football League), and CME presentations (APA, Shire/Takeda, Globala Medical Education, and US Psychiatric Congress). RR is a Senior Epidemiologist at Inflexxion, an IBH Company. Inflexxion contracts with FDA and multiple companies with interests in some of the products included in the compounds evaluated for this article. JG is the Chief Scientific Officer at Inflexxion, an IBH Company. Inflexxion contracts with FDA and multiple companies with interests in some of the products included in the compounds evaluated for this article. JG has served as an expert witness on behalf of two manufacturers of prescription opioid medications.

## Publisher's Note

All claims expressed in this article are solely those of the authors and do not necessarily represent those of their affiliated organizations, or those of the publisher, the editors and the reviewers. Any product that may be evaluated in this article, or claim that may be made by its manufacturer, is not guaranteed or endorsed by the publisher.

## References

[1] Faraone SV Rostain AL Montano CB Mason O Antshel KM Newcorn JH. Systematic review: nonmedical use of prescription stimulants: risk factors, outcomes, and risk reduction strategies. J Am Acad Child Adolesc Psychiatry. (2020) 59:100–12. 31326580 10.1016/j.jaac.2019.06.012

[2] NIDA. Misuse of Prescription Drugs. (2018). Available online at: https://www.drugabuse.gov/publications/research-reports/misuse-prescription-drugs/what-scope-prescription-drug-misuse (accessed August 14, 2019).

[3] Wilens TE Adler LA Adams J Sgambati S Rotrosen J Sawtelle R. Misuse and diversion of stimulants prescribed for ADHD: a systematic review of the literature. J Am Acad Child Adolesc Psychiatry. (2008) 47:21–31. 10.1097/chi.0b013e31815a56f1 18174822

[4] Mattson ME. Emergency Department Visits Involving Attention Deficit/Hyperactivity Disorder Stimulant Medications. The CBHSQ Report. Rockville, MD: The CBHSQ Report: January 24, 2013. Center for Behavioral Health Statistics and Quality, Substance Abuse and Mental Health Services Administration (2013). 27631057

[5] Chen L-Y Crum RM Strain EC Alexander GC Kaufmann C Mojtabai R. Prescriptions, nonmedical use, and emergency department visits involving prescription stimulants. J Clin Psychiatry. (2016) 77:e297–304. 10.4088/JCP.14m09291 26890573 PMC5903919

[6] Cassidy TA Varughese S Russo L Budman SH Eaton TA Butler SF. Nonmedical use and diversion of ADHD stimulants among U.S. Adults Ages 18-49: A National Internet Survey. J Atten Disord. (2012) 19:630–40. 10.1177/1087054712468486 23269194

[7] Benson K Flory K Humphreys KL Lee SS. Misuse of stimulant medication among college students: a comprehensive review and meta-analysis. Clin Child Fam Psychol Rev. (2015) 18:50–76. 10.1007/s10567-014-0177-z 25575768

[8] McCabe SE Teter CJ Boyd CJ. Medical use, illicit use and diversion of prescription stimulant medication. J Psychoactive Drugs. (2006) 38:43–56. 10.1080/02791072.2006.10399827 16681175 PMC1761861

[9] Novak SP Kroutil LA Williams RL Van Brunt DL. The nonmedical use of prescription ADHD medications: results from a national internet panel. Subst Abuse Treat Prev Policy. (2007) 2:32. 10.1186/1747-597X-2-32 17974020 PMC2211747

[10] Weyandt LL Oster DR Marraccini ME Gudmundsdottir BG Munro BA Rathkey ES. Prescription stimulant medication misuse: where are we and where do we go from here? Exp Clin Psychopharmacol. (2016) 24:400–14. 10.1037/pha0000093 27690507 PMC5113141

[11] McCance-Katz EF. The National Survey on Drug Use and Health: 2017. 2017 NSDUH Report America's Behavioral Health Changes & Challenges. (2018). Available online at: https://www.samhsa.gov/data/sites/default/files/nsduh-ppt-09-2018.pdf (accessed February 5, 2019).

[12] Garnier LM Arria AM Caldeira KM Vincent KB O'Grady KE Wish ED. Sharing and selling of prescription medications in a college student sample. J Clin Psychiatry. (2010) 71:262–9. 10.4088/JCP.09m05189ecr 20331930 PMC2845992

[13] Arria AM Caldeira KM O'Grady KE Vincent KB Fitzelle DB Johnson EP. Drug exposure opportunities and use patterns among college students: results of a longitudinal prospective cohort study. Subst Abus. (2008) 29:19–38. 10.1080/08897070802418451 19042196 PMC2614283

[14] Garnier-Dykstra LM Caldeira KM Vincent KB O'Grady KE Arria AM. Nonmedical use of prescription stimulants during college: four-year trends in exposure opportunity, use, motives, and sources. J Am Coll Health. (2012) 60:226–34. 10.1080/07448481.2011.589876 22420700 PMC3313072

[15] DeSantis AD Webb EM Noar SM. Illicit use of prescription ADHD medications on a college campus: a multimethodological approach. J Am Coll Health. (2008) 57:315–24. 10.3200/JACH.57.3.315-324 18980888

[16] Clemow DB Walker DJ. The potential for misuse and abuse of medications in ADHD: a review. Postgrad Med. (2014) 126:64–81. 10.3810/pgm.2014.09.2801 25295651

[17] Arria AM Caldeira KM Vincent KB O'Grady KE Cimini MD Geisner IM. Do college students improve their grades by using prescription stimulants nonmedically? Addict Behav. (2017) 65:245–9. 10.1016/j.addbeh.2016.07.016 27469455 PMC5140739

[18] Arria AM Wilcox HC Caldeira KM Vincent KB Garnier-Dykstra LM O'Grady KE. Dispelling the myth of “smart drugs”: cannabis and alcohol use problems predict nonmedical use of prescription stimulants for studying. Addict Behav. (2013) 38:1643–50. 10.1016/j.addbeh.2012.10.002 23254212 PMC3558594

[19] Arria AM DuPont RL. Nonmedical prescription stimulant use among college students: why we need to do something and what we need to do. J Addict Dis. (2010) 29:417–26. 10.1080/10550887.2010.509273 20924877 PMC2951617

[20] Teter CJ McCabe SE LaGrange K Cranford JA Boyd CJ. Illicit use of specific prescription stimulants among college students: prevalence, motives, and routes of administration. Pharmacotherapy. (2006) 26:1501–10. 10.1592/phco.26.10.1501 16999660 PMC1794223

[21] White BP Becker-Blease KA Grace-Bishop K. Stimulant medication use, misuse, and abuse in an undergraduate and graduate student sample. J Am Coll Health. (2006) 54:261–8. 10.3200/JACH.54.5.261-268 16539218

[22] Compton WM Volkow ND. Major increases in opioid analgesic abuse in the United States: concerns and strategies. Drug Alcohol Depend. (2006) 81:103–7. 10.1016/j.drugalcdep.2005.05.009 16023304

[23] Faraone S V Hess J Wilens T. Prevalence and consequences of the nonmedical use of amphetamine among persons calling poison control centers. J Atten Disord. (2019) 23:1219–28. 10.1177/1087054719843182 31169052

[24] Schiller LJ. The food and drug administration solicits input on potential role for abuse-deterrent formulations of central nervous system stimulants; establishment of a public docket; request for comments. Fed Regist. (2019) 84:49530–5.

[25] Coplan PM Chilcoat HD Butler SF Sellers EM Kadakia A Harikrishnan V. The effect of an abuse-deterrent opioid formulation (OxyContin) on opioid abuse-related outcomes in the postmarketing setting. Clin Pharmacol Ther. (2016) 100:275–86. 10.1002/cpt.390 27170195 PMC5102571

[26] Severtson SG Ellis MS Kurtz SP Rosenblum A Cicero TJ Parrino MW. Sustained reduction of diversion and abuse after introduction of an abuse deterrent formulation of extended release oxycodone. Drug Alcohol Depend. (2016) 168:219–29. 10.1016/j.drugalcdep.2016.09.018 27716575

[27] Simon K Worthy SL Barnes MC Tarbell B. Abuse-deterrent formulations: transitioning the pharmaceutical market to improve public health and safety. Ther Adv drug Saf. (2015) 6:67–79. 10.1177/2042098615569726 25922655 PMC4406920

[28] Ford JA Schepis TS McCabe SE. Poly-prescription drug misuse across the life course: prevalence and correlates across different adult age cohorts in the U.S. Int J Drug Policy. (2021) 88:103017. 10.1016/j.drugpo.2020.103017 33227640 PMC8005409

[29] Kandel D. Stages in adolescent involvement in drug use. Science. (1975) 190:912–4. 10.1126/science.1188374 1188374

[30] Behavioral Health Statistics and Quality. 2018 National Survey on Drug Use and Health Public Use File Codebook. Rockville, MD (2019). Available online at: https://www.datafiles.samhsa.gov/sites/default/files/field-uploads-protected/studies/NSDUH-2018/NSDUH-2018-datasets/NSDUH-2018-DS0001/NSDUH-2018-DS0001-info/NSDUH-2018-DS0001-info-codebook.pdf (accessed August 20, 2020).

[31] FDA. Petition Denial Response Letter to John C. Kulli MD Redacted. (2014). Available online at: https://www.regulations.gov/document?D=FDA-2006-P-0453-0005 (accessed May 23, 2019).

[32] Carlier J Giorgetti R Varì MR Pirani F Ricci G Busardò FP. Use of cognitive enhancers: methylphenidate and analogs. Eur Rev Med Pharmacol Sci. (2019) 23:3–15. 10.26355/eurrev_201901_16741 30657540

[33] Szumilas M. Explaining odds ratios. J Can Acad Child Adolesc Psychiatry. (2010) 19:227–9. 20842279 PMC2938757

[34] Fairman RT Vu M Haardörfer R Windle M Berg CJ. Prescription stimulant use among young adult college students: who uses, why, and what are the consequences? J Am Coll Health. (2020). 10.1080/07448481.2019.1706539. [Epub ahead of print]. 31944915 PMC7363509

[35] Mowlem FD Rosenqvist MA Martin J Lichtenstein P Asherson P Larsson H. Sex differences in predicting ADHD clinical diagnosis and pharmacological treatment. Eur Child Adolesc Psychiatry. (2019) 28:481–9. 10.1007/s00787-018-1211-3 30097723 PMC6445815

[36] Kakuszi B Bitter I Czobor P. Suicidal ideation in adult ADHD: gender difference with a specific psychopathological profile. Compr Psychiatry. (2018) 85:23–9. 10.1016/j.comppsych.2018.06.003 29957374

[37] Kariisa M Scholl L Wilson N Seth P Hoots B. Drug overdose deaths involving cocaine and psychostimulants with abuse potential — United States, 2003–2017. MMWR. (2019) 68:388–95. 10.15585/mmwr.mm6817a3 31048676 PMC6541315

[38] Compton WM Volkow ND. Abuse of prescription drugs and the risk of addiction. Drug Alcohol Depend. (2006) 83(Suppl. 1):S4–7. 10.1016/j.drugalcdep.2005.10.020 16563663

[39] McCabe SE Teter CJ Boyd CJ Wilens TE Schepis TS. Sources of prescription medication misuse among young adults in the United States: the role of educational status. J Clin Psychiatry. (2018) 79:17m11958. 10.4088/JCP.17m11958 29570970 PMC5932281

[40] Weyandt LL Janusis G Wilson KG Verdi G Paquin G Lopes J. Nonmedical prescription stimulant use among a sample of college students: relationship with psychological variables. J Atten Disord. (2009) 13:284–96. 10.1177/1087054709342212 19767596

[41] Sweeney CT Sembower MA Ertischek MD Shiffman S Schnoll SH. Nonmedical use of prescription ADHD stimulants and preexisting patterns of drug abuse. J Addict Dis. (2013) 32:1–10. 10.1080/10550887.2012.759858 23480243 PMC3630453

[42] Schuermeyer J Salomonsen-Sautel S Price RK Balan S Thurstone C Min S-J. Temporal trends in marijuana attitudes, availability and use in Colorado compared to non-medical marijuana states: 2003-11. Drug Alcohol Depend. (2014) 140:145–55. 10.1016/j.drugalcdep.2014.04.016 24837585 PMC4161452

[43] Wilkinson ST Yarnell S Radhakrishnan R Ball SA D'Souza DC. Marijuana legalization: impact on physicians and public health. Annu Rev Med. (2016) 67:453–66. 10.1146/annurev-med-050214-013454 26515984 PMC4900958

[44] Lynskey MT Heath AC Bucholz KK Slutske WS Madden PAF Nelson EC. Escalation of drug use in early-onset cannabis users vs co-twin controls. JAMA. (2003) 289:427–33. 10.1001/jama.289.4.427 12533121

[45] Nkansah-Amankra S Minelli M. “Gateway hypothesis” and early drug use: additional findings from tracking a population-based sample of adolescents to adulthood. Prev Med Rep. (2016) 4:134–41. 10.1016/j.pmedr.2016.05.003 27413674 PMC4929049

[46] Vanyukov MM Tarter RE Kirillova GP Kirisci L Reynolds MD Kreek MJ. Common liability to addiction and “gateway hypothesis”: theoretical, empirical and evolutionary perspective. Drug Alcohol Depend. (2012) 123(Suppl.):S3–17. 10.1016/j.drugalcdep.2011.12.018 22261179 PMC3600369

[47] Dunne EM Hearn LE Rose JJ Latimer WW. ADHD as a risk factor for early onset and heightened adult problem severity of illicit substance use: an accelerated gateway model. Addict Behav. (2014) 39:1755–8. 10.1016/j.addbeh.2014.07.009 25123341 PMC4180292

[48] McCabe SE Dickinson K West BT Wilens TE. Age of onset, duration, and type of medication therapy for attention-deficit/hyperactivity disorder and substance use during adolescence: a multi-cohort National study. J Am Acad Child Adolesc Psychiatry. (2016) 55:479–86. 10.1016/j.jaac.2016.03.011 27238066 PMC4921895

[49] Frati P Kyriakou C Del Ri A Marinelli E Vergallo GM Zaami S. Smart drugs and synthetic androgens for cognitive and physical enhancement: revolving doors of cosmetic neurology. Curr Neuropharmacol. (2015) 13:5–11. 10.2174/1570159X13666141210221750 26074739 PMC4462043

[50] DEA. Prevention With Purpose: A Strategic Planning Guide for Preventing Drug Misuse Among College Students. (2020). Available online at: https://www.campusdrugprevention.gov/sites/default/files/Strategic Planning Guide (Final-Online) (1).pdf (accessed February 6, 2020).

[51] Faraone S V Wilens TE Petty C Antshel K Spencer T Biederman J. Substance use among ADHD adults: implications of late onset and subthreshold diagnoses. Am J Addict. (2007) 16(Suppl. 1):24–32; quiz 33–4. 10.1080/10550490601082767 17453604

[52] Newcorn JH Weiss M Stein MA. The complexity of ADHD: diagnosis and treatment of the adult patient with comorbidities. CNS Spectr. (2007) 12(8 Suppl. 12):1–14; quiz 15–6. 10.1017/S1092852900026158 17667893

[53] Musso MW Gouvier WD. “Why is this so hard?” A review of detection of malingered ADHD in college students. J Atten Disord. (2014) 18:186–201. 10.1177/1087054712441970 22582347

[54] Ramsay JR Rostain AL. The Adult ADHD Tool Kit: Using CBT to Facilitate Coping Inside and Out. New York, NY: Routledge (2015).

[55] Conrod PJ Castellanos-Ryan N Strang J. Brief, personality-targeted coping skills interventions and survival as a non-drug user over a 2-year period during adolescence. Arch Gen Psychiatry. (2010) 67:85–93. 10.1001/archgenpsychiatry.2009.173 20048226

[56] Buchanan T LeMoyne T. Helicopter parenting and the moderating impact of gender for university students with ADHD. Int J Disabil Dev Educ. (2020) 67:18–27. 10.1080/1034912X.2019.1634794

